# Investigation on physical properties and modification mechanisms of diatomite/SBR modified asphalt

**DOI:** 10.1371/journal.pone.0286328

**Published:** 2023-09-28

**Authors:** Di Li, Rui Chen

**Affiliations:** College of Environment and Civil Engineering, Chengdu University of Technology, Chengdu, Sichuan, China; Shandong University of Technology, CHINA

## Abstract

In recent years, diatomite has been successfully adopted in asphalt modification to overcome the problems of polymer modified asphalt, because of the advantages in wide sources, low price, and worthy technical characteristics. Although the improvement of the high-temperature performance of the modified diatomite asphalt has been verified in previous studies, the diatomite will bring negative impact on the low-temperature resistance. Hence, the objective of this study is to seek a new channel to improve the comprehensive performance of the diatomite modified asphalt binder. Considering the advantage of the SBR in improving the low-temperature performance of asphalt binder, the diatomite/SBR composite modified asphalt binder (DSA) and the corresponding preparation technology are developed to obtain an improved comprehensive performance via the orthogonal experiment method in this study. Moreover, the modification mechanisms of the DSA are revealed using fluorescence microscopy (FM) tests, Gel permeation chromatography (GPC) tests, and Fourier transform infrared spectroscopy (FTIR) tests.

## 1. Introduction

By the end of 2020 in China, the length of highways had reached 161,000 km, of which more than 90% are asphalt pavement [1–3]. Obviously, the asphalt binder plays an important role in the performance of the asphalt pavement. To achieve this target, polymers, such as styrene-butadiene-styrene (SBS) [4], Styrene butadiene rubber (SBR) [5], polyethylene (PE) [6], and ethylene-vinyl-acetate (EVA) [7], have been widely adopted to improve the engineering performance of asphalt binder. However, the existing modifiers are difficult to get a balance among performance, economy, and technological complexity [8–10]. Therefore, it is necessary to consider using new materials to improve the comprehensive performance of modified asphalt.

Diatomite is a kind of biogenic siliceous sedimentary rock and mainly composed of silica. Owing to the advantages in wide sources, low price, and worthy technical characteristics (e.g., high hardness, high porosity, strong adsorption capacity, and good stability) [11, 12], the diatomite has been applied in asphalt modification. For instance, Song et al. [13] and Cong et al. [14] found that as an asphalt modifier, diatomite has been proved to significantly improve the conventional indexes of asphalt binder, and improve its high-temperature stability and anti-aging performance to varying degrees. Yang et al. [15] and Shukry et al. [16] found that the addition of diatomite significantly improved the high temperature performance of asphalt mixture, and its fatigue performance and water stability were significantly better than that of the base asphalt mixture. However, with the increase of diatomite content, its low-temperature performance may be damaged. For instance, Luo et al. [17] found that when the diatomite content exceeds 13%, the low temperature performance of the diatomite modified asphalt is lower than that of the base asphalt due to the system is often unstable and the ductility at 15°C is decreased significantly.

Therefore, there have been some studies focusing on diatomite composite modified to improve the comprehensive performance of the diatomite modified asphalt binder. For example, Liu et al. [18, 19] found that the softening point, viscosity and needle penetration index of the diatomite composite rubber powder (RP) modified asphalt increased with the increase of diatomite content, while needle penetration and ductility decreased. Liang et al. [20] found that with the addition of RP and diatomite increased, the ability of instantaneous elastic deformation, viscoelastic deformation, irreversible permanent deformation, and the relaxation modulus of the diatomite/RP composite modified asphalt mixtures can be improved.

The previous studies are mainly focused on the diatomite/RP composite modified asphalt binder. However, they are not able to tackle the lack of the low-temperature resistance of diatomite modified. Hence, the objective of this study is to seek a new channel to improve the comprehensive performance of the diatomite modified asphalt binder. From the above, it can be understood that the improvement of high temperature performance of diatomite is due to the addition of diatomite itself to absorb the easy flowing and small molecular weight aromatic and saturated fractions in asphalt, but also causes the imbalance of the four components of asphalt [17]. SBR modified asphalt, which is also based on physical modification, is precisely by reducing the bee structure in the asphalt, that is, reducing the content of asphaltenes to achieve the effect of improving low temperature [33]. Therefore, SBR should be able to complement the characteristics of diatomite modified asphalt with superior high temperature performance and general low temperature performance, and composite modification can obtain modified asphalt with excellent comprehensive performance. Above all, this study plans to investigate and prepare diatomite /SBR modified asphalt by combining various factors through orthogonal tests. Moreover, the modification mechanisms of the DSA are revealed using fluorescence microscopy (FM) tests, Gel permeation chromatography (GPC) tests, and Fourier transform infrared spectroscopy (FTIR) tests.

The list of abbreviations which used in the following text, as shown in Table 1.

**Table 1:** The list of abbreviations.

Expansion	Abbreviations
Diatomite/SBR composite modified asphalt	DSA
Styrene butadiene rubber	SBR
Fluorescence microscopy	FM
Gel permeation chromatography	GPC
Fourier transform infrared spectroscopy	FTIR

## 2. Materials and methods

### 2.1. Materials

**Fig 1:**
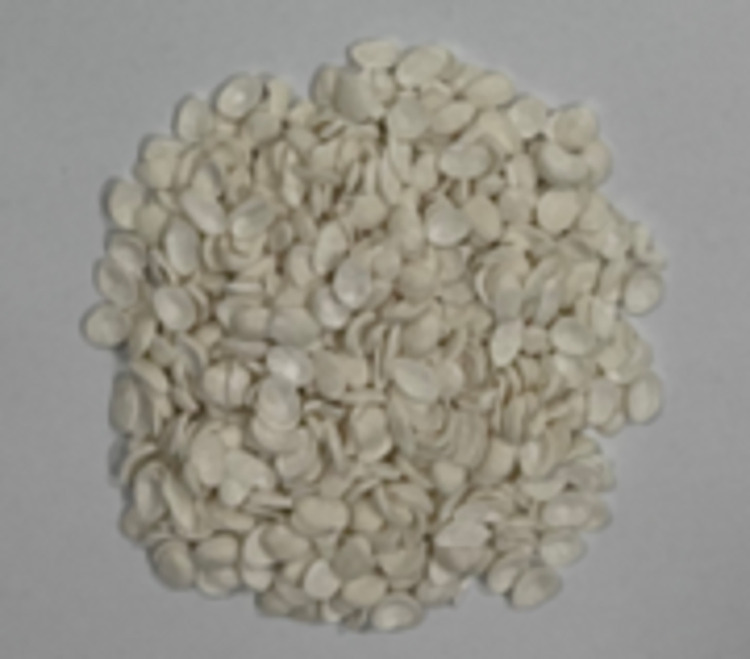
SBR asphalt modifier.

**Fig 2:**
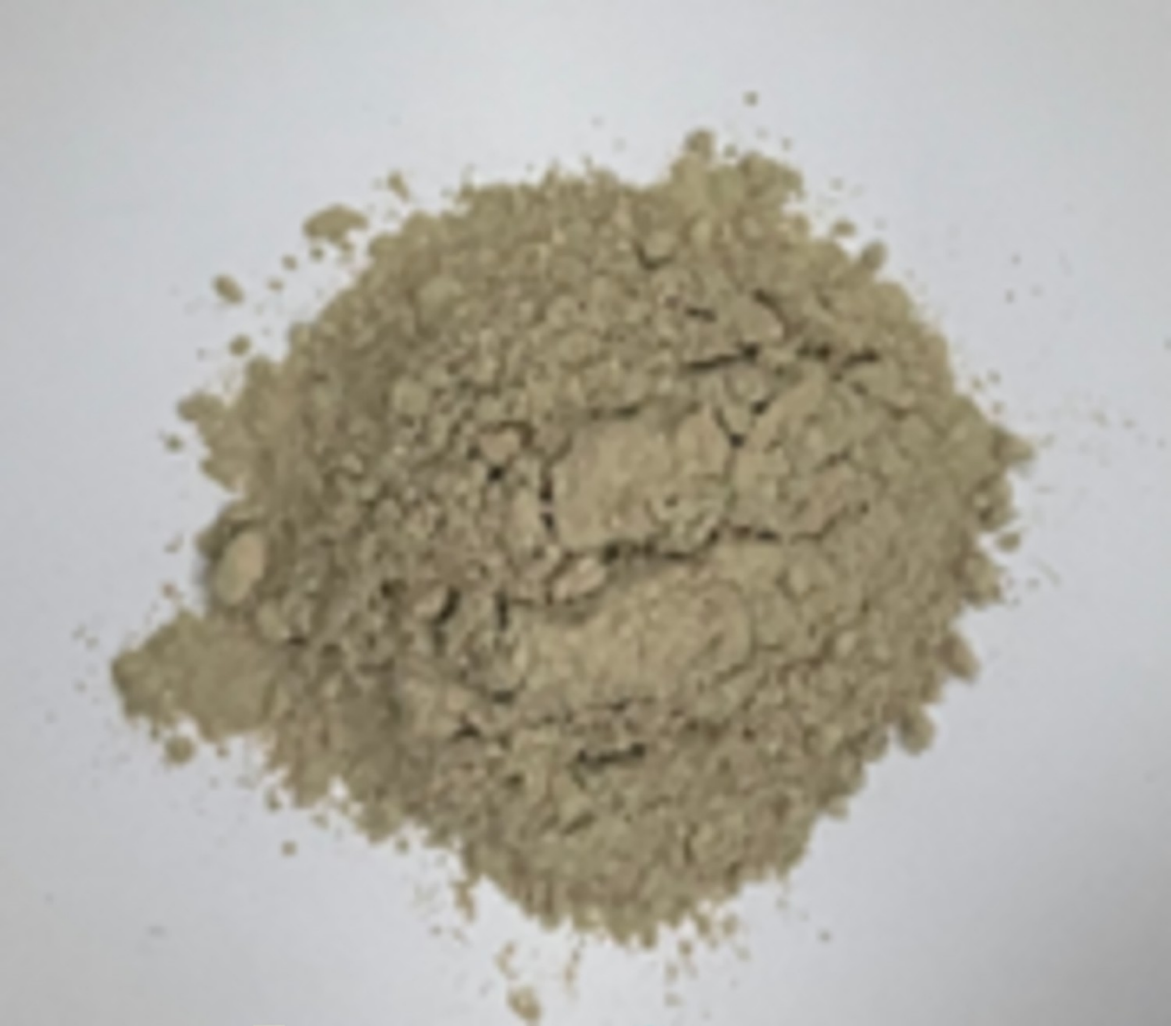
Diatomite.

In this study, Donghai 90# base asphalt binder, 90# Polymerized Styrene Butadiene Rubber (SBR) asphalt modifier, and diatomite were mixed to produce the DSA. Their technical indexes are presented in Tables 2–4. The SBR asphalt modifier used in this study is a type of white granular material manufactured from Shandong Shuanghan Petrochemical Equipment Co., Ltd in Fig 1. The diatomite is obtained from Shengzhou in Zhejiang province of China, is a kind of soil yellow powder manufactured from silicone sedimentary rock, as shown in Fig 2.

**Table 2:** Technical indexes of base asphalt binder.

penetration at 25°C (0.1mm)	Softening point (°C)	ductility at 10°C (cm)	ductility at 15°C (cm)	Viscosity at 60°C (Pa·s)
84	46	>100	>100	178

**Table 3:** Technical indexes of SBR asphalt modifier.

Volatile content (%)	Ash content (%)	Organic acid (%)	Bound styrene (%)	Tensile strength (145°C 35min) (Mpa)	Elongation (145°C 35min) (%)
≤0.90	≤0.50	4.50–6.75	22.50–24.50	≥24.50	≥330

**Table 4:** Technical indexes of diatomite.

Mineral composition	SiO_2_	AL_2_O_3_	Fe_2_O_3_	CaO	MgO
Content ratio (%)	75	5.5	15	1.0	1.25

### 2.2. Methods

#### (1) Preparation of the DSA

The DSA is prepared according to the following steps.

- Heat the base asphalt to an oven at 140°C until it has good fluidity.
- Mix the SBR and diatomite with the base asphalt and stir to swell for 20 minutes.
- According to the set shear temperature, shear number rate and shear time to complete the shear mixing.
- After the shearing was completed, the development was stirred at 160°C for 3 hours at 600 r·min^-1^ to complete the preparation of the DSA.

#### (2) Conventional performance test of asphalt

**Fig 3:**
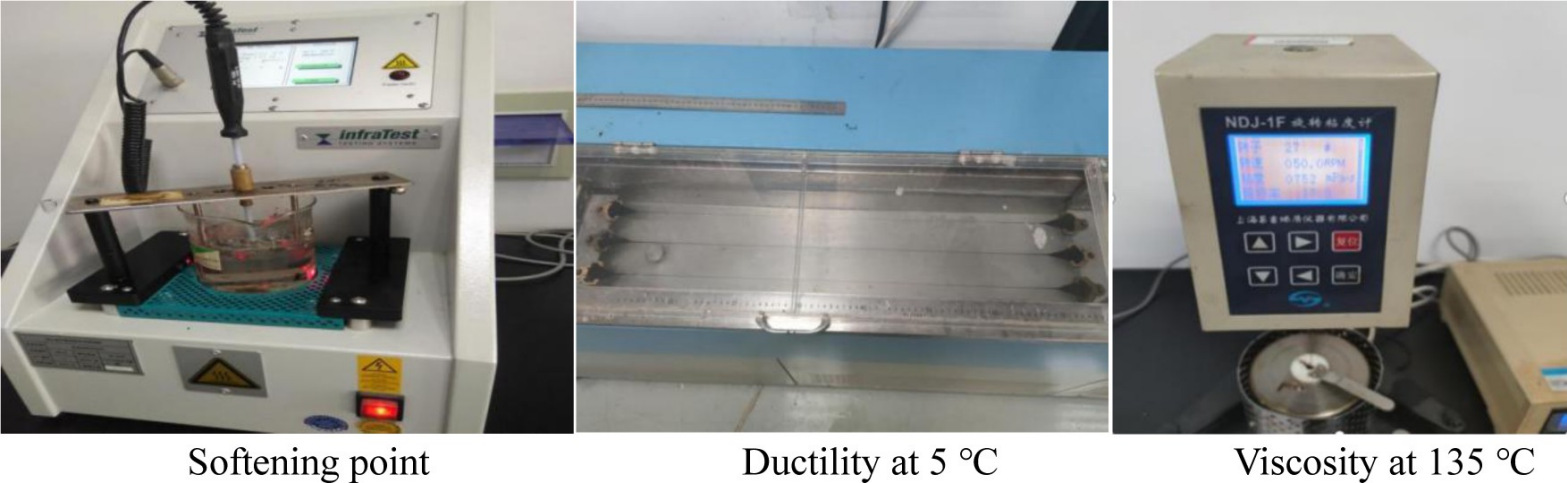
Conventional performance test.

Softening point reflects the temperature sensitivity of asphalt, ductility at 5°C reflects the plasticity ability of asphalt at low temperatures, Brinell viscosity at 135°C reflects the flow resistance of asphalt at high temperatures. These three performance indicators can well characterize the high and low temperature performance of asphalt at the macro level. The softening point, ductility at 5°C and Brinell viscosity at 135°C tests of asphalt shown in the Fig 3 are carried out according to T0606, T0605 and T0625 in *Test Regulations for Asphalt and Asphalt Mixture in Highway Engineering* (JTGE20-2011) (Ministry of Transport, 2011).

#### (3) Fourier Transform (FTIR) test

**Fig 4:**
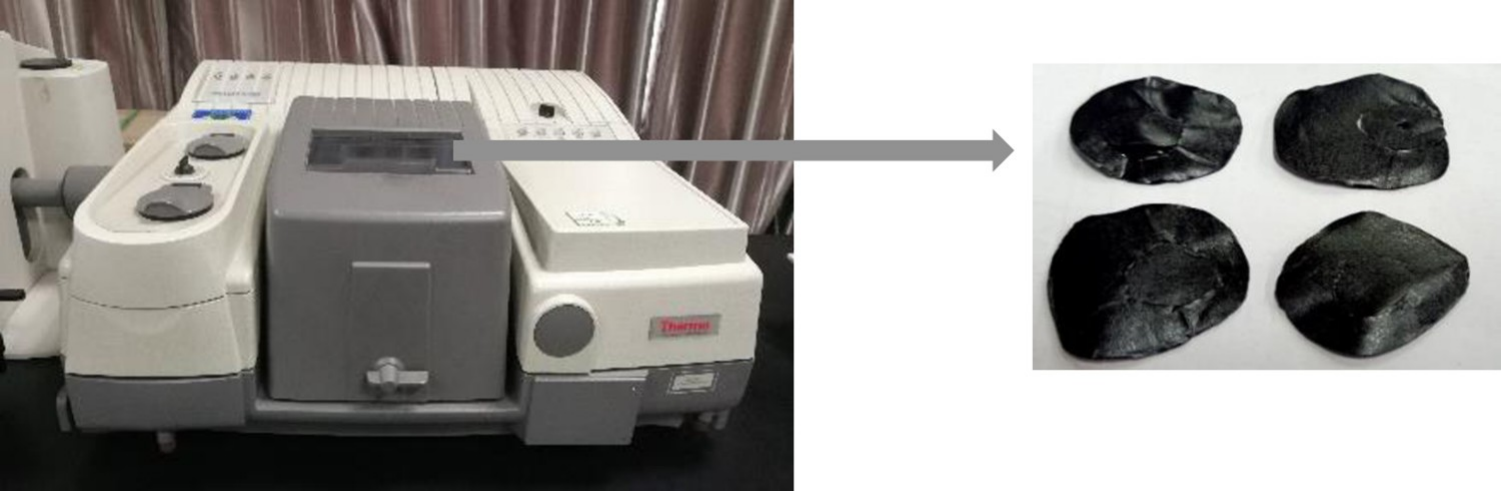
FTIR.

The Nicolet 5700 Fourier transform infrared spectrometer as shown in Fig 3 is adopted in this study, which is able to investigate the functional groups of asphalt-based materials [21, 22]. The spectral range is 400–4000 cm^-1^, and the resolution is 0.4 cm^-1^. The FTIR sample of the asphalt binder is approximatively circular disc with the diameter of 1–1.5 cm, as shown in Fig 4.

#### (1) Gel permeation chromatography (GPC) test

**Fig 5:**
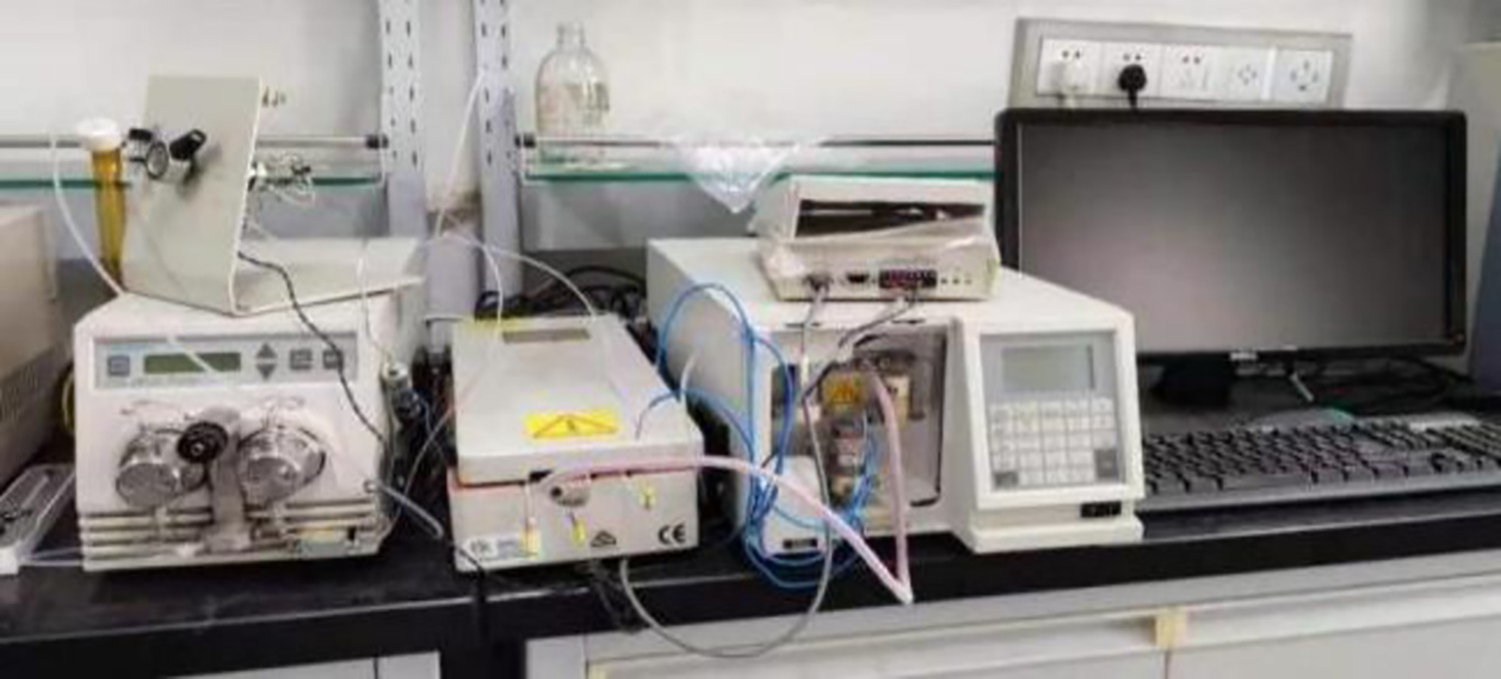
GPC.

The Waters 515–410 gel permeation chromatograph as shown in Fig 5 is adopted in this study, which is able to investigate the molecular weight size and distribution of substances of the asphalt. The addition of the modifier causes a change in the molecular weight within the asphalt. GPC test makes the test sample flow through the column filled with gel, and then separates according to the penetration degree of solute molecules of different sizes in the gel column [23–26].

#### (2) Fluorescence microscopic (FM) test

**Fig 6:**
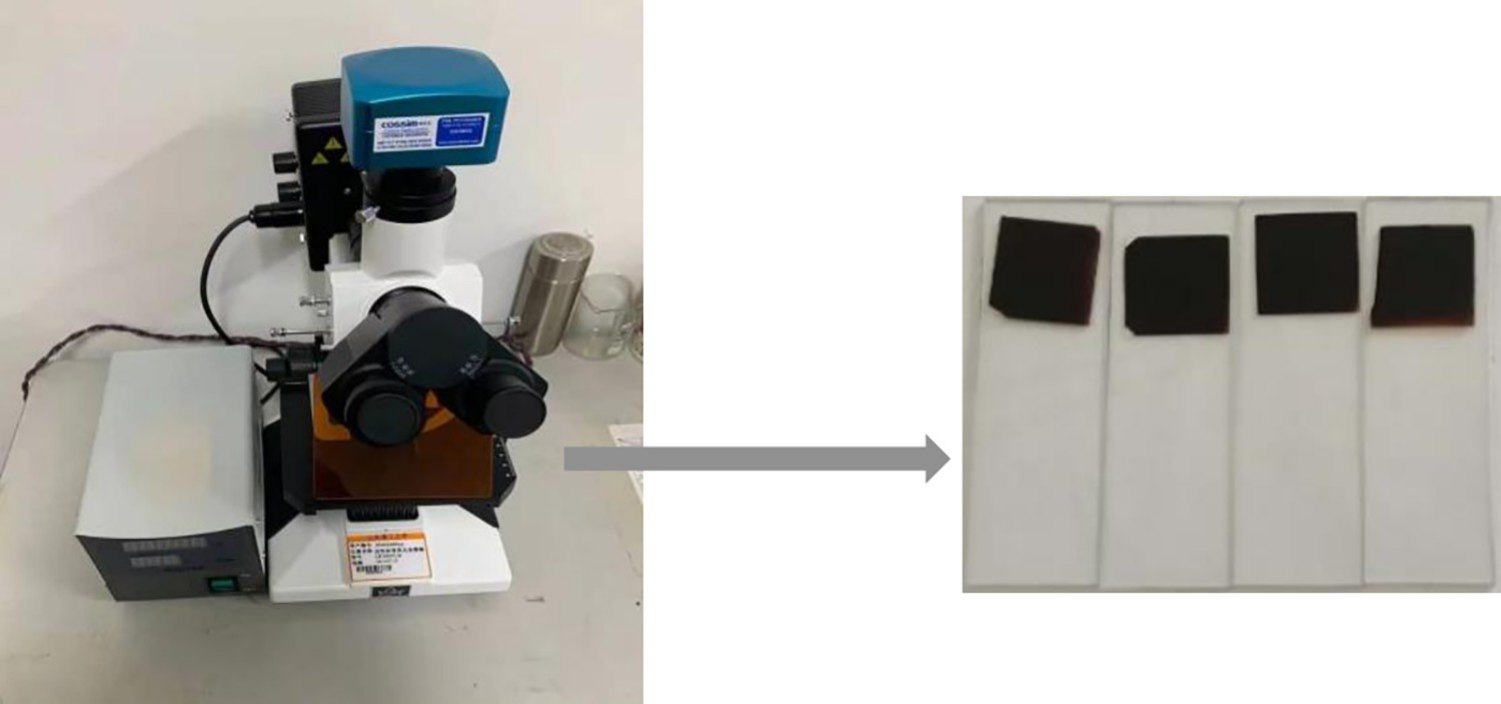
FM.

The LW100 FT/B fluorescence microscope as shown in Fig 6 is adopted in this study, which is able to stimulate the fluorescent substance in asphalt-based materials to fluoresce under the action of ultraviolet light. Accordingly, the microscopic structure of the asphalt-based materials can be observed at different levels of magnification [27–31]. The preparation of FM sample of the asphalt is as follows.

- Heat the asphalt binders and the glass slide. The asphalt binders must achieve a flow state. The surface temperature of the glass slide should be 150–160°C.
- Drip 1–2 g asphalt onto the heated glass slide, and put the cover glass down from one side of the glass slide quickly and gently.
- After the asphalt on the glass slide is completely covered by the cover glass, gently rock the glass slide to distribute the asphalt evenly. The FM samples of the asphalt can be obtained after the glass slide is cooled for 30 min, as shown in Fig 6.

## 3. Conventional performance analysis

The softening point, ductility at 5°C and Brinton viscosity at 135°C of the DSA are mainly affected by five factors: diatomite content, SBR content, shear temperature, shear rate and shear time. In order to cut the test work scientifically and reveal the complex relevance of these factors, the orthogonal test method is adopted in this study. The factors and levels of the orthogonal test are shown in Table 5, and the orthogonal test scheme and results are shown in Table 6.

**Table 5:** Factors and levels of the orthogonal test.

Level	Factor				
	A	B	C	D	E
	Diatomite content (%)	SBR content (%)	Shear temperature (°C)	Shear rate (r•min^-1^)	Shear time (min)
1	3	1	150	3000	20
2	6	1.5	160	3500	30
3	9	2	170	4000	40
4	12	2.5	180	4500	50
5	15	3	190	5000	60

**Table 6:** Scheme and results of the orthogonal test.

Test number	A	B	C	D	E	Softening point (°C)	ductility at 5°C (cm)	viscosity at 135°C (Pa·s)
1	3	1	150	3000	20	55.0	23.6	0.61
2	3	1.5	160	3500	30	55.5	37.0	0.64
3	3	2	170	4000	40	56.0	45.1	0.73
4	3	2.5	180	4500	50	56.4	49.0	0.75
5	3	3	190	5000	60	57.0	47.0	0.81
6	6	1	160	4000	50	57.2	23.0	0.56
7	6	1.5	170	4500	60	58.8	33.0	0.70
8	6	2	180	5000	20	57.2	35.0	0.71
9	6	2.5	190	3000	30	58.0	38.0	0.81
10	6	3	150	3500	40	57.6	43.5	0.88
11	9	1	170	5000	30	57.8	19.3	0.71
12	9	1.5	180	3000	40	57.2	22.0	0.61
13	9	2	190	3500	50	59.0	30.4	0.78
14	9	2.5	150	4000	60	60.0	40.0	0.95
15	9	3	160	4500	20	59.0	43.2	0.95
16	12	1	180	3500	60	59.0	14.8	0.79
17	12	1.5	190	4000	20	58.0	26.3	0.74
18	12	2	150	4500	30	59.0	33.0	0.83
19	12	2.5	160	5000	40	58.8	35.2	1.19
20	12	3	170	3000	50	59.5	37.0	1.15
21	15	1	190	4500	40	58.5	8.0	0.83
22	15	1.5	150	5000	50	58.2	13.0	0.83
23	15	2	160	3000	60	59.3	14.7	0.90
24	15	2.5	170	3500	20	59.0	20.1	0.92
25	15	3	180	4000	30	60.8	23.8	1.12

As shown in Table 6, it can be found that the Brinell viscosity at 135°C are far less than 3, which meets the requirement of *Technical Specifications for Construction of Highway Asphalt Pavement* (JTG F40-2004) (Ministry of Transport, 2004). Therefore, only the softening point and ductility at 5°C are analyzed in the follow-up. The range method is used to analyze the range value of each factor on the performance of the DSA. A higher range of one factor represents a higher influence degree of the factor. The meanings of computational symbols *K*_*i*_, *k*_*i*_ and *R* are as follows.

- *K*_*i*_ represents the sum of the corresponding test results when the level number on any column is *i*.
- *k*_*i*_ = *K*_*i*_ / *S*, *S* is the frequency of occurrence of each level in any column.
- Range *R* = max {*k*_*1*_, *k*_*2*_, *k*_*3*_}- min {*k*_*1*_, *k*_*2*_, *k*_*3*_} on any of the columns.

### 3.1. Softening point

**Fig 7:**
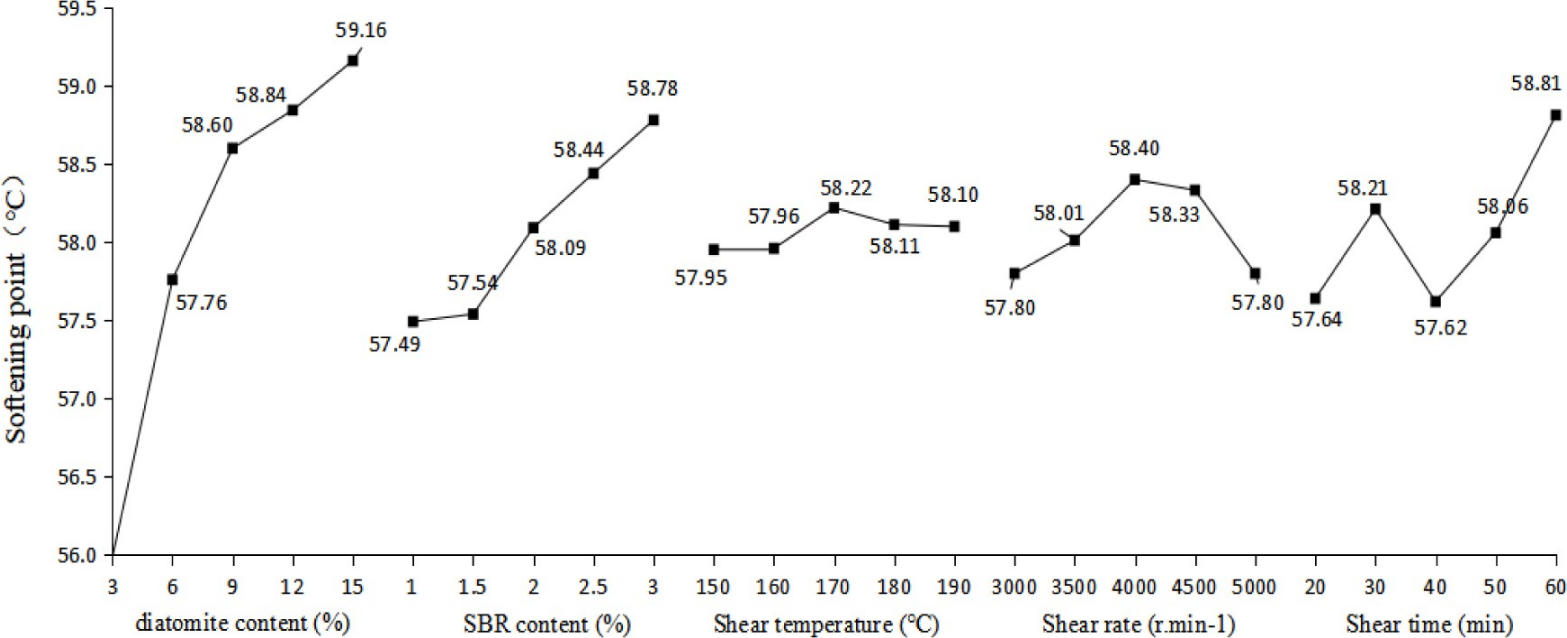
Trends of softening point.

Analysis of the softening point range results based on orthogonal test is shown in Table 7 and Fig 7.

**Table 7:** Analysis of softening point range results.

Conventional performance	Level	A		B	C	D	E
Softening point (°C)	*K*_*1*_	279.9		287.5	289.8	289.0	288.2
	*K*_*2*_	288.8		287.7	289.8	290.1	291.1
	*K*_*3*_	293.0		290.5	291.1	292.0	288.1
	*K*_*4*_	294.2		292.2	290.6	291.7	290.3
	*K*_*5*_	295.8		293.9	290.5	289.0	294.1
	*k*_*1*_	56.0		57.5	58.0	57.8	57.6
	*k*_*2*_	57.8		57.5	58.0	58.0	58.2
	*k*_*3*_	58.6		58.1	58.2	58.4	57.6
	*k*_*4*_	58.8		58.4	58.1	58.3	58.1
	*k*_*5*_	59.2		58.8	58.1	57.8	58.8
	*R*	3.2		1.3	0.3	0.6	1.2
Factors sequence			A > B > E > D > C				

As shown in Table 7 and Fig 7, in general, it can be found that the influence degree of each factor on the softening point of the DSA is diatomite content > SBR content > Shear time > Shear rate > Shear temperature. As can be seen from the above table and Fig, with the increase of the content of diatomite and SBR, the softening point of modified asphalt is on the rise. When the content of diatomite increased to 9%, it tended to be flat. When the content of SBR increased to 2.5%, the softening point reached the peak, and the softening point increased by 4.6% and 2.2%, respectively. This phenomenon may be attributed to the absorption of saturated and aromatic components in asphalt components by diatomite, which may be greater than the effect of SBR addition on asphalt components. With the increase of shear temperature, shear rate and shear time, the softening point of modified asphalt fluctuates up and down to different degrees, and the fluctuation range is not large, and the influence on the softening point is not obvious. When the content of diatomite is 9 ~ 15%, the content of SBR is 2.5 ~ 3%, the shear temperature is 170 ~ 190°C, the shear rate is 4500 r·min^-1^, and the shear time is 50 ~ 60 min, the softening point of the DSA is better.

### 3.2. 5°C ductility

**Fig 8:**
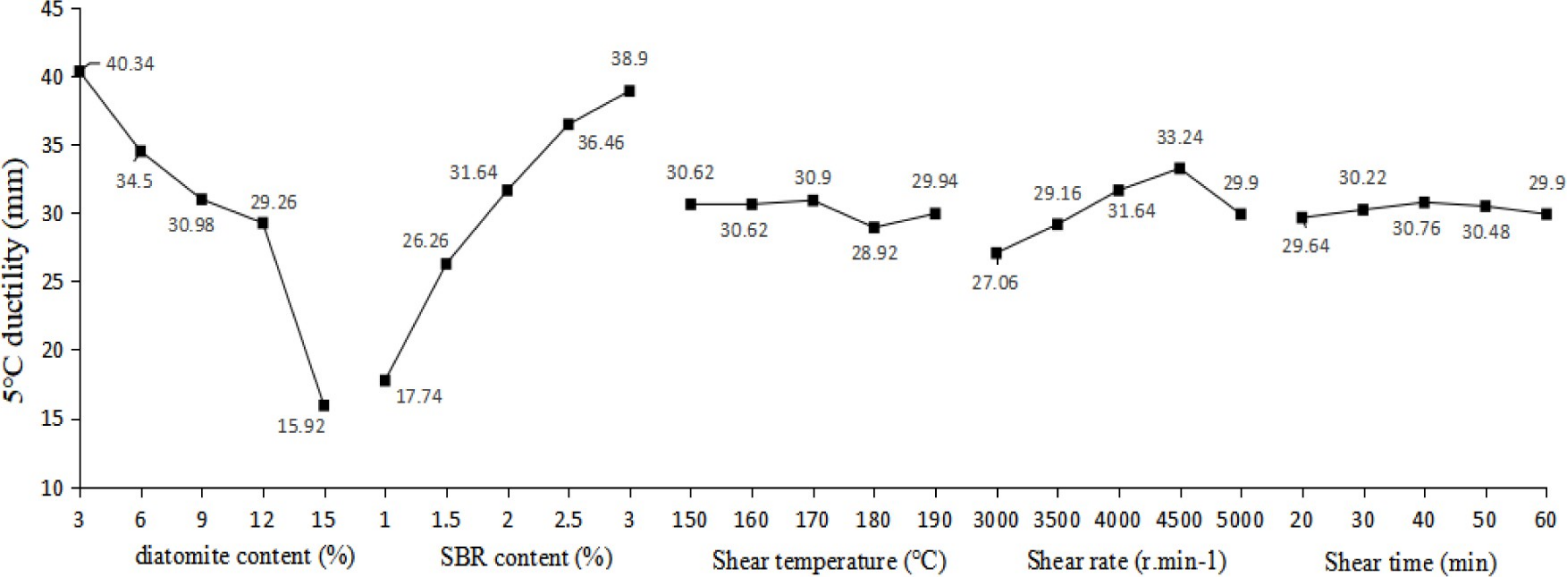
Trends of ductility at 5°C.

Analysis of the ductility at 5°C range results based on orthogonal test is shown in Table 8 and Fig 8.

**Table 8:** Ranges of ductility at 5°C.

Conventional performance	Level	A	B	C	D	E
Ductility at 5°C (cm)	*K*_*1*_	201.7	88.7	153.1	135.3	148.2
	*K*_*2*_	172.5	131.3	153.1	145.8	151.1
	*K*_*3*_	154.9	158.2	154.5	158.2	153.8
	*K*_*4*_	146.3	182.3	144.6	166.2	152.4
	*K*_*5*_	79.6	194.5	149.7	149.5	149.5
	*k*_*1*_	40.3	17.7	30.6	27.1	29.6
	*k*_*2*_	34.5	26.3	30.6	29.2	30.2
	*k*_*3*_	31.0	31.6	30.9	31.6	30.8
	*k*_*4*_	29.3	36.5	28.9	33.2	30.5
	*k*_*5*_	15.9	38.9	29.9	29.9	29.9
	*R*	24.4	21.2	2.0	6.2	1.1
Factors sequence		A > B > D > C > E				

As shown in Table 8 and Fig 8, it can be found that the influence degree of each factor on the ductility at 5°C of the DSA is diatomite content > SBR content > shear rate > shear temperature > shear time. According to the above Table 8 and Fig 8, with the increase of diatomite content, the ductility of modified asphalt shows a decreasing trend, and the curve trend changes obviously before and after the content of 12%. With the increase of SBR content, the ductility of modified asphalt shows a rising trend. When the SBR content is 3%, the ductility of modified asphalt is greatly increased by 119%. This phenomenon shows that the addition of SBR does improve the equilibrium of the four components of asphalt with a small amount of diatomite, which ensures the stability of the asphalt system to a certain extent. However, the complementarity of SBR to the disadvantages of diatomite modified asphalt was not significant at diatomaceous earth doping above 12%. Modified asphalt ductility with the increase of shear temperature, shear rate and shear time, the overall trend of change is consistent, the change is not large, there is a peak phenomenon. When the content of diatomite is equal to 3 ~ 9%, the content of SBR is equal to 2.5 ~ 3%, the shear temperature is 160 ~ 170°C, the shear rate is 4500 r·min^-1^, and the shear time is 40 ~ 50 min, the ductility at 5°C of the DSA is better.

### 3.3. Discussion

In conclusion, the content of diatomite and SBR has a significant effect on the softening point and ductility at 5°C of the DSA, while the effects of the shear rate, shear temperature, and shear time are relatively limited. Therefore, combined with the above analysis results, it is planned to select the diatomite content, SBR content, shear temperature, shear rate and shear time as 6 ~ 12%, 2.5 ~ 3%, 170°C, 4500 r·min^-1^, and 50 min to carry out the conventional performance test of the DSA (the results are shown in Table 9) to further optimize the preparation process.

**Table 9:** Conventional performance.

Test number	A (%)	B (%)	C (°C)	D(r•min^-1^)	E(min)	Ductility at 5°C (cm)	Softening point (°C)	Viscosity at 135°C (Pa.s)
26	6	2.5	170	4500	50	44.5	58.8	0.95
27	9	2.5	170	4500	50	42.4	60.2	1.02
28	12	2.5	170	4500	50	36.1	61	1.18
29	6	3	170	4500	50	45.7	59.5	0.98
30	9	3	170	4500	50	43.5	60.8	1.05
31	12	3	170	4500	50	37	61.8	1.21

As shown in Table 9, it can be found that when the content of SBR increases from 2.5% to 3%, the softening point, the ductility at 5°C, and the Brinton viscosity at 135°C only on average increase by 2.6%, 1.2%, and 2.9%, respectively. Hence, the content of the SBR is selected to be 2.5% from a view of economic performance. When the content of diatomite increases from 6% to 9% and from 9% to 12%, the ductility at 5°C decreases by 5.1% and 17.6%, respectively. This is due to that the viscosity of the asphalt increases and its fluidity decreases with the addition of the diatomite, and the low-temperature brittleness increases accordingly. With the increase of diatomite content, the diatomite will be irregularly dispersed and stacked in asphalt, causing that the internal cementation of the asphalt is weaken and the low-temperature performance will further decrease. According to the requirements of the ductility at 5°C of the SBR modified asphalt in the *Technical Specifications for Construction of Highway Asphalt Pavement* (JTG F40-2004), the principle of the highest softening point is taken as the premise that the ductility at 5°C is greater than 40cm, and the final diatomite content is 9%. Therefore, the optimal blending scheme is proposed as follows: the diatomite content is 9%, the SBR content is 2.5%, the shear temperature is 170°C, the shear rate is 4500 r•min^-1^, the shear time is 50 min. The DSA has superior and stable performance.

The subsequent the DSA refers to No.27 in Table 9. In order to verify the merits and disadvantages of the DSA, the performance of composite modified asphalt is compared with the base asphalt, 9% diatomite modified asphalt and 5% SBS modified asphalt through softening point, ductility at 5°C, and Brinton viscosity at 135°C. The results are shown in Table 9.

As shown in Table 10, it can be found that the softening points of the SBS modified asphalt, the diatomite modified asphalt, and the DSA are 40.7%, 26.1%, and 16.1% higher than the base asphalt, respectively. The Brinton viscosities at 135°C are 336.1%, 66.7%, and 183.3% higher, respectively. Moreover, the ductilities at 5°C of the SBS modified asphalt and the DSA has been improved significantly, especially for the DSA. The ductility at 5°C of the DSA is 36.6% higher than the SBS modified asphalt, showing the advantage of the DSA in the low-temperature performance.

**Table 10:** Comparison of conventional performance of asphalt.

Conventional performance	Base asphalt	SBS modified asphalt	Diatomite modified asphalt	DSA
Softening point (°C)	46.0	64.7	58.0	60.2
Ductility at 5°C (cm)	1	30.0	0	42.4
Viscosity at 135°C (Pa·s)	0.36	1.57	0.85	1.02

## 4. Compound modification mechanism analysis

### 4.1. FM test

**Fig 9:**
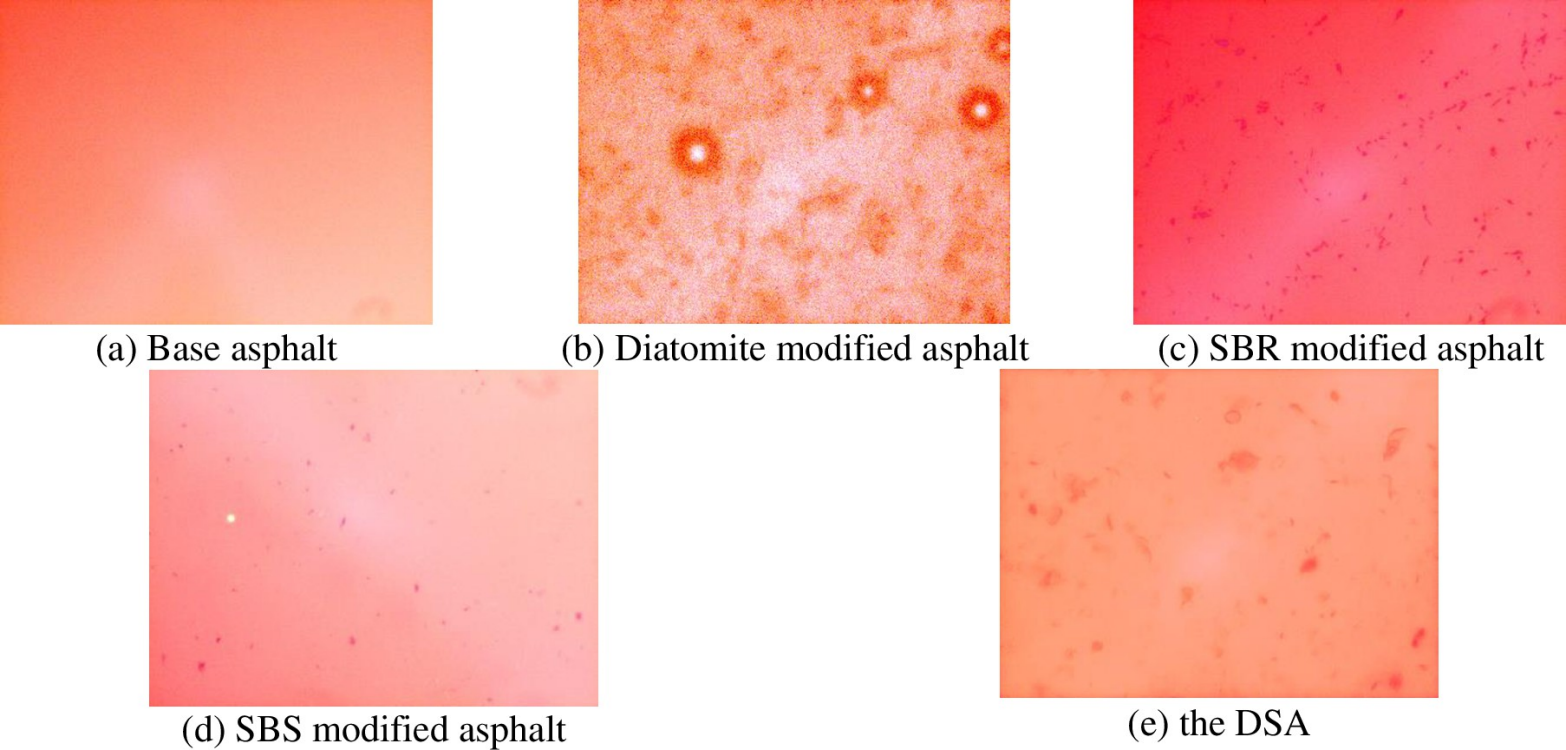
Fluorescent micrograph of asphalt.

The FM images of the base asphalt, diatomite modified asphalt, SBR modified asphalt, SBS modified asphalt and DSA magnified 100 time are shown in Fig 9.

As shown in Fig 9(A), it can be found that under fluorescence microscope, the base asphalt is a continuous phase. When the diatomite is added separately (see Fig 9(B)), the diatomite will cluster in the base asphalt to form a relatively large size, which is not evenly distributed to establish a good network structure. As shown in Fig 9(D), it can be found that there are few particles with large particle size in the SBS phase, which are evenly distributed in the base asphalt and tend to develop into two-phase continuous. This is because the modifier particles absorb the components of the base asphalt and expand, and this structure is basically the ideal structure of polymer modified asphalt [32]. As can be shown in Fig 9(C), it can be found that after mixing and shearing of the SBR and base asphalt, the SBR particles are further cut into tiny particles, but the dispersion effect of the SBR is not ideal. Compared with the SBS modified asphalt, the compatibility of the SBR phase and base asphalt is obviously worse than the SBS [33]. The base asphalt is a continuous phase and polymer is a dispersed phase of the single-phase flocculent continuous structure. When the diatomite is added into the SBR modified asphalt (see Fig 9(E)), compared with the single diatomite modified asphalt or the SBR modified asphalt, the micro-fine distribution of the DSA is better than that of single modified asphalt. The modifier particles are smaller and evenly distributed as a whole, forming a certain network structure with the base asphalt. The physical properties of the DSA are better than those of the diatomite modified asphalt and SBR modified asphalt.

### 4.2. GPC test

**Fig 10:**
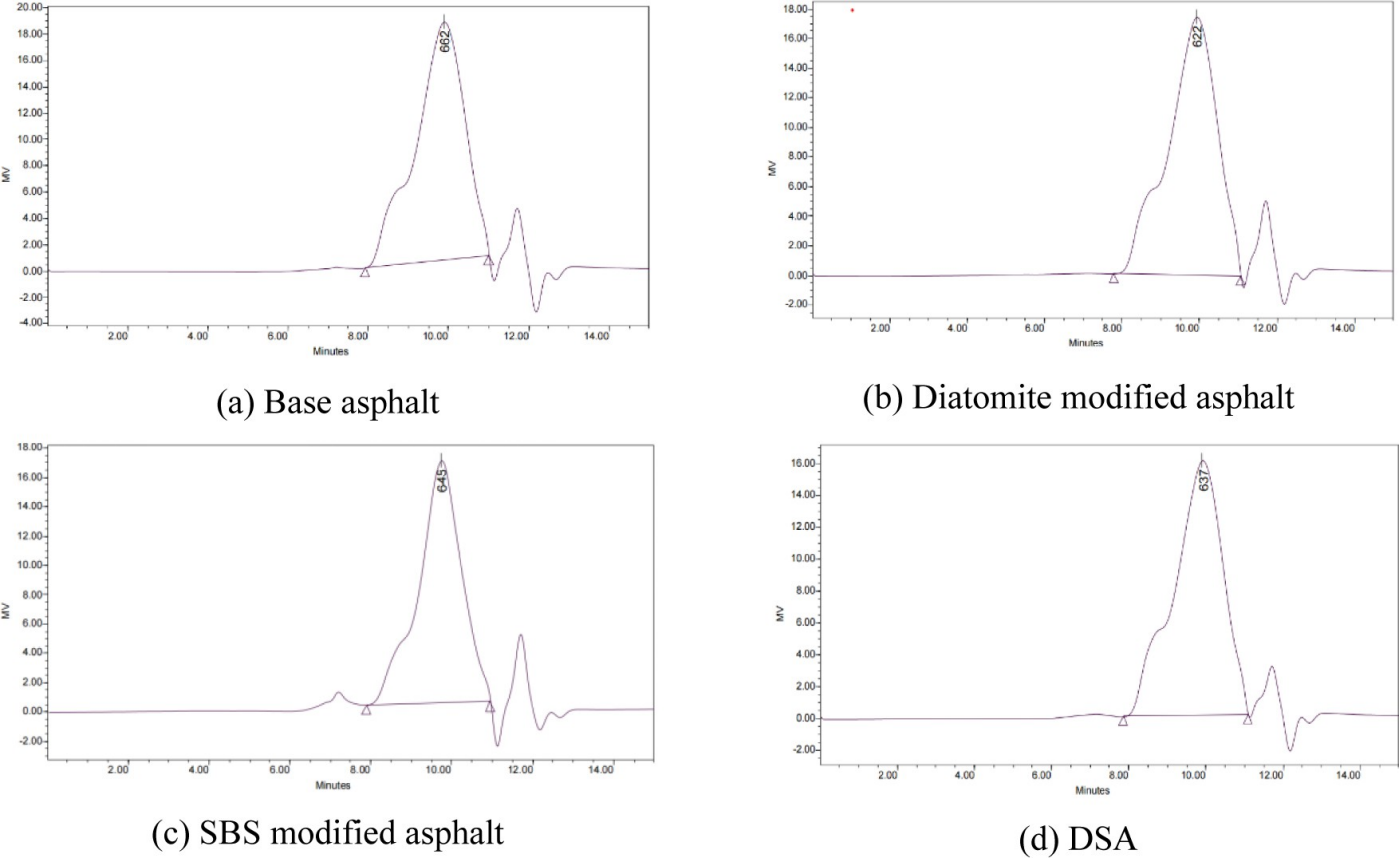
GPC chromatogram.

The GPC chromatogram and test results of the base asphalt, diatomite modified asphalt, SBS modified asphalt and the DSA are presented in Fig 10 and Table 11. The numerically average molecular weight Mn (the average statistical molecular weight based on the number of molecules), the weight average molecular weight Mw (the average statistical molecular weight based on the molecular weight) and the polydispersity coefficient *d*, as expressed in Eqs (1), (2), and (3), respectively.


$$M_{n}=\frac{\sum N_{i}M_{i}}{\sum N_{i}}$$ (1)

$$M_{W}=\frac{\sum N_{i}W_{i}}{\sum N_{i}}$$ (2)

$$d=\frac{M_{W}}{M_{n}}$$ (3)

Where *N*_i_ is the number of molecules with molecular weight *M*_i_, *W*_i_ is the weight of the component with molecular weight *M*_i_.

**Table 11:** GPC test results.

Species	Base asphalt	Diatomite modified asphalt	SBS modified asphalt	DSA
Weight-average molecular weight (*M*_w_)	1840	1851	1875	1866
Number-average molecular weight (*M*_n_)	594	562	566	569
Polydispersity coefficient (*d* = *M*_w_/*M*_n_)	3.096	3.309	3.312	3.280
Peak molecular weight (*M*_p_)	662	622	645	637

As shown in Fig 10 and Table 11, it can be found that the peak molecular weight *M*_p_ of the diatomite modified asphalt, SBS modified asphalt, and DSA are smaller than that of base asphalt. This indicates that the incorporation of the diatomite modifier, SBS modifier and Diatomite/SBR composite modifier decreases the content of small molecular weight components in asphalt, while the phase pair of large molecular weight components increases, so the interaction between molecules increases, which makes the high-temperature stability of the modified asphalt better than that of the base asphalt.

As shown the data of *M*_W_ in Table 11, it can be found that compared with the base asphalt, the *M*_W_ of the diatomite modified asphalt, SBS modified asphalt and DSA increases by 5% and 6% respectively, which indicating that the shear failure resistance of the diatomite modified asphalt, SBS modified asphalt and DSA are all enhanced. Among them, the DSA and SBS modified asphalt have the best high temperature stability.

As shown the data of *d* in Table 11, it can be found that the polydispersity coefficients *d* of diatomite modified asphalt, the SBS modified asphalt and DSA are all greater than those of base asphalt. The molecular weight distribution becomes more dispersed with the increase of polydispersity coefficient *d*. In other words, the components in a certain range of molecular weight are fewer. Considering the phase transition interval of molecular weight is consistent with the temperature interval of the transformation of asphalt aggregation components, the heat absorption of asphalt binder (i.e., the temperature sensitivity of asphalt binder) will be reduced.

### 4.3. FTIR test

**Fig 11:**
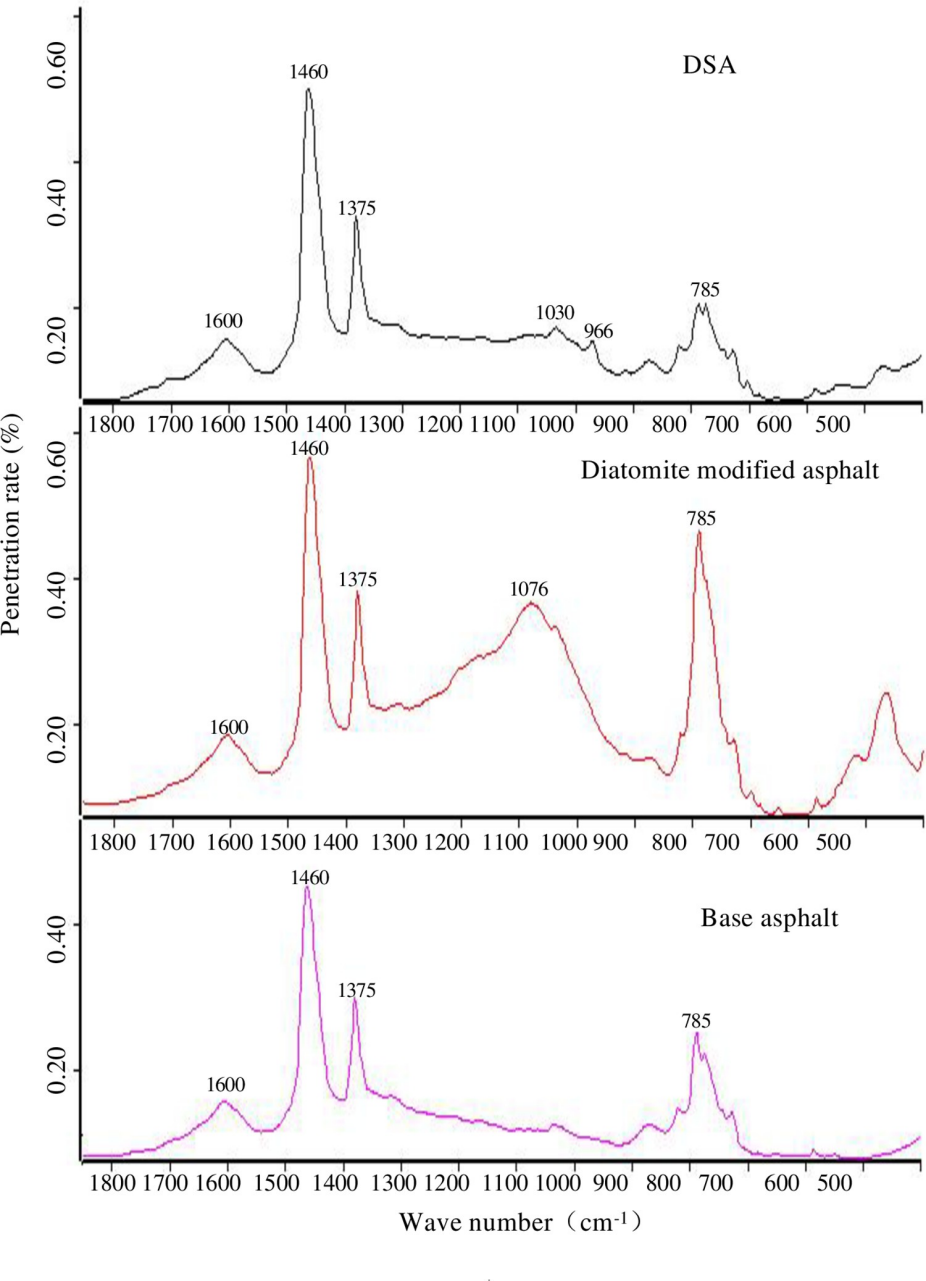
IR spectra of modified asphalt and base asphalt.

In order to further explore the influence of the composite modifier on asphalt, the functional groups of the base asphalt, diatomite modified asphalt and the DSA via the FTIR tests, as shown in Fig 11.

As shown in Fig 11, it can be observed the absorption peaks at 1600 cm^-1^, 1460 cm^-1^, 1375 cm^-1^ and 785 cm^-1^ in all the spectrograms. The absorption peaks at 1600 cm^-1^, 1460 cm^-1^ and 1375 cm^-1^ are caused by the stretching of the C = C in benzene ring, the stretching of the CH_3_, deformation of the CH_3_, and the stretching of the S = O (sulfoxide), respectively. The absorption peaks at 785 cm^-1^ are caused by the rocking of the -CH_2_ in benzene ring of polystyrene.

Moreover, the appearance of S = O stretching vibration absorption peak near 1076cm^-1^ is the only difference between the IR spectrums of the base asphalt and diatomite modified asphalt, which is proving the existence of the diatomite. Therefore, the addition of diatomite does not produce new functional groups, but only a physical blending process. Compared with base asphalt, the IR spectrum of the DSA generates two new characteristic peaks near 1030 cm^-1^ and 966cm^-1^. The absorption peak near 1030cm^-1^ should be the stretching of the S = O (sulfoxide) vibration absorption peak of diatomite, and the absorption peaks at 966 cm^-1^ is caused by the wagging of the trans-C-H-disubstituted -CH = CH_2_ in styrene-butadiene, which should be the characteristic absorption peak of trans-butadiene of the SBR. The generation of the S = O will increase the intermolecular forces. The corresponding macroscopic performance presents the increase of hardness and the shear failure resistance of the diatomite modified asphalt and the DSA.

In general, the absorption peak of the DSA is the same as that of the base asphalt. It can be determined that the chemical composition and relative content of the DSA and the base asphalt are basically similar, and when the diatomite and SBR are added to the base asphalt at the same time, it is mainly physical blending, and no complex chemical reaction occurs.

## 5. Conclusions

This study investigated the performance of the DSA via the orthogonal experiment method, and revealed the modification mechanisms of the DSA by FM tests, GCP tests, and FTIR tests. It greatly expands the applicability of SBR modified asphalt, and this experiment provides a reference for future research on dosage and blending method through complicated orthogonal test. On the other hand, through the micro-level verification of the modification effect of diatomite, it also provides technical support for promoting the wide use of this cheap material. Based on the findings of this study, the following conclusions can be drawn.

1. The optimal preparation parameters of the DSA are put forward as follows: shear temperature is 170°C, shear rate is 4500 r·min^-1^, and shear time is 50 min.
2. The content of diatomite and SBR have a significant effect on the ductility at 5°C and the softening point of the DSA. When the content of diatomite and SBR is equal to 9% and 2.5%, respectively, the comprehensive performance of the DSA is appropriate.
3. The modified particles are small and evenly distributed in the DSA, and form a certain network structure with the base asphalt. Compared with the base asphalt, the average molecular weight and polydispersity coefficient of the DSA increased, which means that the DSA has good shear deformation resistance and low temperature sensitivity. And only two vibration absorption peaks in the infrared spectrum are different from base asphalt, and the overall trend is consistent. The comprehensive performance of the DSA is improved by physical blending of diatomite and SBR.
4. Finally, the research on the pavement performance of diatomite /SBR modified asphalt combined aggregate remains to be carried out. The composite modified asphalt prepared in this test is based on the conventional performance index of asphalt as the standard, so the dosage and mixing conditions obtained are only applicable to the road conditions under general and ordinary environmental conditions. In the case of complex load or extreme conditions, the reference index should be appropriately adjusted according to the situation. Secondly, due to the porous structure of diatomite, it should have great research potential in the future to improve the aging properties of asphalt materials.

## Supporting information

S1 File This file includes all the experiment data. (DOCX)
