# Supplementary material for: Investigation on physical properties and modification mechanisms of diatomite/SBR modified asphalt
Source: PLoS One. 2023 Sep 28;18(9):e0286328. doi: 10.1371/journal.pone.0286328 (PMC10538715; doi:10.1371/journal.pone.0286328)
Supplement: S1 File — (DOCX) [file pone.0286328.s001.docx]

**Factors and levels of the orthogonal test**

| Level | Factor | | | | |
| --- | --- | --- | --- | --- | --- |
|  | A | B | C | D | E |
|  | Diatomite content  (%) | SBR content  (%) | Shear temperature  (℃) | Shear rate  (r•min^-1^) | Shear time  (min) |
| 1 | 3 | 1 | 150 | 3000 | 20 |
| 2 | 6 | 1.5 | 160 | 3500 | 30 |
| 3 | 9 | 2 | 170 | 4000 | 40 |
| 4 | 12 | 2.5 | 180 | 4500 | 50 |
| 5 | 15 | 3 | 190 | 5000 | 60 |

**Scheme and results of the orthogonal test**

| Test number | A | B | C | D | E | Softening point (℃) | ductility at 5 ℃ (cm) | viscosity at 135 ℃ (Pa·s) |
| --- | --- | --- | --- | --- | --- | --- | --- | --- |
| 1 | 3 | 1 | 150 | 3000 | 20 | 55.0 | 23.6 | 0.61 |
| 2 | 3 | 1.5 | 160 | 3500 | 30 | 55.5 | 37.0 | 0.64 |
| 3 | 3 | 2 | 170 | 4000 | 40 | 56.0 | 45.1 | 0.73 |
| 4 | 3 | 2.5 | 180 | 4500 | 50 | 56.4 | 49.0 | 0.75 |
| 5 | 3 | 3 | 190 | 5000 | 60 | 57.0 | 47.0 | 0.81 |
| 6 | 6 | 1 | 160 | 4000 | 50 | 57.2 | 23.0 | 0.56 |
| 7 | 6 | 1.5 | 170 | 4500 | 60 | 58.8 | 33.0 | 0.70 |
| 8 | 6 | 2 | 180 | 5000 | 20 | 57.2 | 35.0 | 0.71 |
| 9 | 6 | 2.5 | 190 | 3000 | 30 | 58.0 | 38.0 | 0.81 |
| 10 | 6 | 3 | 150 | 3500 | 40 | 57.6 | 43.5 | 0.88 |
| 11 | 9 | 1 | 170 | 5000 | 30 | 57.8 | 19.3 | 0.71 |
| 12 | 9 | 1.5 | 180 | 3000 | 40 | 57.2 | 22.0 | 0.61 |
| 13 | 9 | 2 | 190 | 3500 | 50 | 59.0 | 30.4 | 0.78 |
| 14 | 9 | 2.5 | 150 | 4000 | 60 | 60.0 | 40.0 | 0.95 |
| 15 | 9 | 3 | 160 | 4500 | 20 | 59.0 | 43.2 | 0.95 |
| 16 | 12 | 1 | 180 | 3500 | 60 | 59.0 | 14.8 | 0.79 |
| 17 | 12 | 1.5 | 190 | 4000 | 20 | 58.0 | 26.3 | 0.74 |
| 18 | 12 | 2 | 150 | 4500 | 30 | 59.0 | 33.0 | 0.83 |
| 19 | 12 | 2.5 | 160 | 5000 | 40 | 58.8 | 35.2 | 1.19 |
| 20 | 12 | 3 | 170 | 3000 | 50 | 59.5 | 37.0 | 1.15 |
| 21 | 15 | 1 | 190 | 4500 | 40 | 58.5 | 8.0 | 0.83 |
| 22 | 15 | 1.5 | 150 | 5000 | 50 | 58.2 | 13.0 | 0.83 |
| 23 | 15 | 2 | 160 | 3000 | 60 | 59.3 | 14.7 | 0.90 |
| 24 | 15 | 2.5 | 170 | 3500 | 20 | 59.0 | 20.1 | 0.92 |
| 25 | 15 | 3 | 180 | 4000 | 30 | 60.8 | 23.8 | 1.12 |

**Analysis of softening point range results**

| Conventional performance | Level | A | | B | C | D | E |
| --- | --- | --- | --- | --- | --- | --- | --- |
| Softening point (℃) | *K_1_* | 279.9 | | 287.5 | 289.8 | 289.0 | 288.2 |
|  | *K_2_* | 288.8 | | 287.7 | 289.8 | 290.1 | 291.1 |
|  | *K_3_* | 293.0 | | 290.5 | 291.1 | 292.0 | 288.1 |
|  | *K_4_* | 294.2 | | 292.2 | 290.6 | 291.7 | 290.3 |
|  | *K_5_* | 295.8 | | 293.9 | 290.5 | 289.0 | 294.1 |
|  | *k_1_* | 56.0 | | 57.5 | 58.0 | 57.8 | 57.6 |
|  | *k_2_* | 57.8 | | 57.5 | 58.0 | 58.0 | 58.2 |
|  | *k_3_* | 58.6 | | 58.1 | 58.2 | 58.4 | 57.6 |
|  | *k_4_* | 58.8 | | 58.4 | 58.1 | 58.3 | 58.1 |
|  | *k_5_* | 59.2 | | 58.8 | 58.1 | 57.8 | 58.8 |
|  | *R* | 3.2 | | 1.3 | 0.3 | 0.6 | 1.2 |
| Factors sequence | | | A > B > E > D > C | | | | |

**Ranges of** **ductility at 5 ℃**

| Conventional performance | Level | A | B | C | D | E |
| --- | --- | --- | --- | --- | --- | --- |
| Ductility at 5 ℃  (cm) | *K_1_* | 201.7 | 88.7 | 153.1 | 135.3 | 148.2 |
|  | *K_2_* | 172.5 | 131.3 | 153.1 | 145.8 | 151.1 |
|  | *K_3_* | 154.9 | 158.2 | 154.5 | 158.2 | 153.8 |
|  | *K_4_* | 146.3 | 182.3 | 144.6 | 166.2 | 152.4 |
|  | *K_5_* | 79.6 | 194.5 | 149.7 | 149.5 | 149.5 |
|  | *k_1_* | 40.3 | 17.7 | 30.6 | 27.1 | 29.6 |
|  | *k_2_* | 34.5 | 26.3 | 30.6 | 29.2 | 30.2 |
|  | *k_3_* | 31.0 | 31.6 | 30.9 | 31.6 | 30.8 |
|  | *k_4_* | 29.3 | 36.5 | 28.9 | 33.2 | 30.5 |
|  | *k_5_* | 15.9 | 38.9 | 29.9 | 29.9 | 29.9 |
|  | *R* | 24.4 | 21.2 | 2.0 | 6.2 | 1.1 |
| Factors sequence | | A > B > D > C > E | | | | |

**Conventional performance**

| Test number | A (%) | B (%) | C (℃) | D(r•min^-1^) | E(min) | Ductility at 5 ℃ (cm) | Softening point (℃) | Viscosity at 135 ℃ (Pa.s) |
| --- | --- | --- | --- | --- | --- | --- | --- | --- |
| 26 | 6 | 2.5 | 170 | 4500 | 50 | 44.5 | 58.8 | 0.95 |
| 27 | 9 | 2.5 | 170 | 4500 | 50 | 42.4 | 60.2 | 1.02 |
| 28 | 12 | 2.5 | 170 | 4500 | 50 | 36.1 | 61 | 1.18 |
| 29 | 6 | 3 | 170 | 4500 | 50 | 45.7 | 59.5 | 0.98 |
| 30 | 9 | 3 | 170 | 4500 | 50 | 43.5 | 60.8 | 1.05 |
| 31 | 12 | 3 | 170 | 4500 | 50 | 37 | 61.8 | 1.21 |

**Comparison of conventional performance of asphalt**

| Conventional performance | Base asphalt | SBS modified asphalt | Diatomite modified asphalt | DSA |
| --- | --- | --- | --- | --- |
| Softening point (℃) | 46.0 | 64.7 | 58.0 | 60.2 |
| Ductility at 5 ℃ (cm) | 1 | 30.0 | 0 | 42.4 |
| Viscosity at 135 ℃ (Pa·s) | 0.36 | 1.57 | 0.85 | 1.02 |

**GPC test results**

| Species | Base asphalt | Diatomite modified asphalt | SBS modified asphalt | DSA |
| --- | --- | --- | --- | --- |
| [Weight-average](javascript:;) [molecular](javascript:;) [weight](javascript:;) (*M*_w_) | 1840 | 1851 | 1875 | 1866 |
| [Number-average](javascript:;) [molecular](javascript:;) [weight](javascript:;) (*M*_n_) | 594 | 562 | 566 | 569 |
| Polydispersity coefficient (*d*=*M*_w_/*M*_n_) | 3.096 | 3.309 | 3.312 | 3.280 |
| Peak molecular weight (*M*_p_) | 662 | 622 | 645 | 637 |
